# Peri-procedural Trans-esophageal Echocardiographic Sizing of the Native Left Ventricular Outflow Tract During Edwards INTUITY Valve Implantation

**DOI:** 10.3389/fcvm.2021.690752

**Published:** 2021-07-15

**Authors:** Kevin Lim, Yan Kit Ho, Simon Chi Ying Chow, Takuya Fujikawa, Alex Pui-Wai Lee, Randolph Hung Leung Wong

**Affiliations:** ^1^Division of Cardiothoracic Surgery, Prince of Wales Hospital, Shatin, Hong Kong; ^2^Division of Cardiology, Department of Medicine, The Chinese University of Hong Kong, Shatin, Hong Kong

**Keywords:** rapid deployment AVR, conduction disturbance, over-sizing, PPM, aortic annulus, LVOT

## Abstract

**Background:** The Edwards INTUITY rapid deployment valve was anchored on the left ventricular outflow tract (LVOT) by radial force akin to transcatheter balloon-expandable valves. This design feature facilitates minimally invasive and complex procedures but comes at the price of compressing the atrioventricular conduction bundle and potential requirement for pacemaker implantation.

**Methods:** A retrospective observational study was conducted on 30 consecutive patients who received the INTUITY valve at our institution from August 2018 to January 2021. Demographical, clinical, and echocardiographic parameters were collected for 90 days post-operatively. The diameter of the native LVOT at the landing site of the sub-annular stent was retrospectively measured using archived trans-esophageal echocardiographic images. A line was drawn from the inner edge of the septal endocardium to the inner edge of the anterior mitral leaflet in mid-systole, parallel to the aortic annulus, 6–8 mm apical to the aortic annulus depending on the valve size and the corresponding stent length. Risk factors associated with new onset conduction disturbances, defined as the occurrence of bundle branch block or complete heart block, were analyzed.

**Results:** Operative mortality was 3.3%. Pre-operatively, permanent pacemakers were required for two patients who were excluded from the subsequent analysis. New onset conduction disturbances occurred in four of the remaining 28 patients (14.3%). This included two incidences of persistent left bundle branch block and two incidences of permanent pacemaker implantation due to complete heart block. Univariate analysis identified over-sizing of the native LVOT by 5 mm or more as a significant risk factor associated with conduction disturbance.

**Conclusion:** During INTUITY vale implantation, in addition to the aortic annulus, the landing site of the sub-annular stent within the native LVOT should also be sized pre-bypass. Over-sizing the native LVOT by 5 mm or more was associated with an increased risk of new onset conduction disturbances and should be avoided.

## Introduction

With ever-expanding indications for transcatheter aortic valve replacement (TAVR), cardiac surgeons face increasing pressure to innovate and improve outcomes after surgical aortic valve replacement (SAVR) (1). One of the most promising innovations in SAVR is the Edwards INTUITY rapid deployment valve system (Edwards Lifesciences LLC, Irvine, California, USA).

The INTUITY valve system consists of a PERIMOUNT Magna Ease (Edwards Lifesciences, Irvine, California, USA) bovine pericardial valve mounted on a crimped, cloth-covered balloon-expandable stainless steel stent, which is solely anchored on the left ventricular outflow tract (LVOT). Unlike TAVR, the valve is implanted after debridement of LVOT calcium; in addition, the depth of valve implantation is pre-determined by the frame length of the chosen valve size.

Large prospective studies in the US and Europe consistently demonstrated reduction in deployment and crossclamp time and improved hemodynamic performance compared with traditional SAVR. Despite debridement of annular calcium, conduction disturbance remains the Achilles' heel of the INTUITY valve. In the TRANSFORM trial, the permanent pacemaker (PPM) rate at 30 days was 13.6%, much higher than that of traditional SAVR at 3–6% (2, 3). The mechanism of conduction disturbance was postulated to be direct compression of the conduction bundle by the sub-annular stent itself.

In this study, we attempted to ascertain the role of peri-procedural trans-esophageal echocardiographic (TEE) guidance in sizing the aortic root and facilitating INTUITY valve implantation. Clinical and echocardiographic risk factors associated with new onset post-operative conduction disturbances were analyzed.

## Materials and Methods

### Study Design

This was a single-center retrospective review of 30 consecutive patients who received the INTUITY valve from August 2018 to January 2021. Patient solicitation was based on patient risk profile, complexity of the operation and surgeon preference. This study was authorized by the Joint Chinese University of Hong Kong – New Territories East Cluster Clinical Research Ethics Committee and consent was waived in view of its retrospective nature.

### Pre-procedural TEE for Sizing the Aortic Annulus

**Figure 1:**
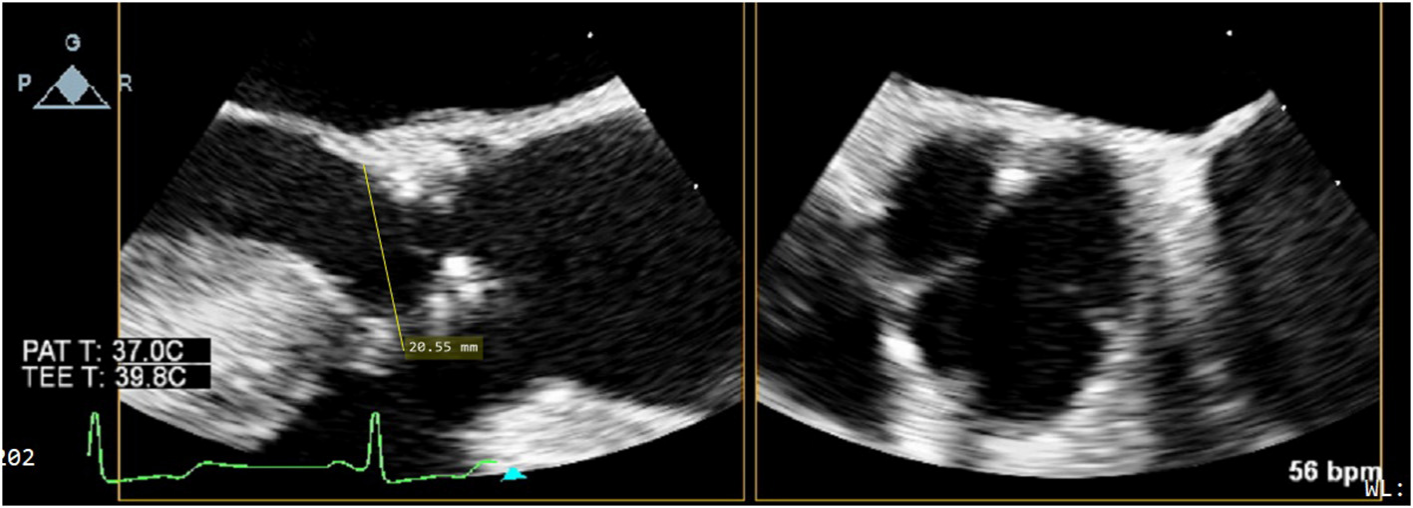
Xplane mode facilitates measurement of aortic annular diameter, especially in the presence of dense LVOT calcium, as is often the case in severe degenerative aortic stenosis.

Prior to institution of cardiopulmonary bypass, the diameter of the aortic annulus and LVOT was measured by two-dimensional TEE with Xplane mode in the mid-esophageal long and short axis views by accredited cardiac anesthesiologists (Figure 1). The plane that bisected the right coronary cusp hinge point and the inter-leaflet triangle between the left and non-coronary cusps posteriorly was used to measure the aortic annulus. In the presence of annular calcium, the plane that bisected the largest diameter but excludes the calcification was used. The relevant images were saved for retrospective analysis.

### The Use of Valve Sizers

After aortotomy, native leaflets were excised, and annular calcium was debrided. The annulus was then sized as per manufacturer instructions using a combined intra- and supra-annular technique. The largest diameter barrel end that fits comfortably in the annulus is chosen, ensuring that the lip of the barrel does not pass through the annulus. The lip of the barrel represents the sewing cuff of the valve; therefore, it is intended to rest on the annulus and not go through it. The replica end of the same sizer was then used to verify adequate fit and orientation of the supra-annular section of the valve in the aortic root.

Detailed nominal dimensions of the Edwards INTUITY valve were listed in Table 1. Of note, the nominal size of the valve corresponds to the internal diameter of the sub-annular stent. The maximum external diameter of the sub-annular stent equals to the nominal size plus 2.5 mm.

**Table 1:** Nominal dimensions of the INTUITY valve.

**Nominal** **size**	**Valve internal** **diameter (mm)**	**Stent internal** **diameter (mm)**	**Stent external** **diameter (mm)**	**Frame** **length (mm)**	***n***	**%**
19	18.0	19.0	21.5	6.0	2	6.7
21	20.0	21.0	23.5	6.6	8	26.7
23	22.0	23.0	25.5	7.2	10	33.3
25	24.0	25.0	27.5	8.0	5	16.7
27	26.0	27.0	29.5	8.0	5	16.7

Three guiding sutures were placed through the nadir of each coronary cusp and then in the corresponding positions of the sewing ring. The valve and delivery system were then lowered onto the annulus and secured into position with snares. The balloon catheter was inflated to deploy the stent. The delivery system was removed, and the three guiding sutures were tied. Finally, the aortotomy was closed.

The final choice of valve size only relied on intraoperative measurements using valve sizers.

### Post-operative TEE to Confirm Valve Position and Absence of PVL

**Figure 2:**
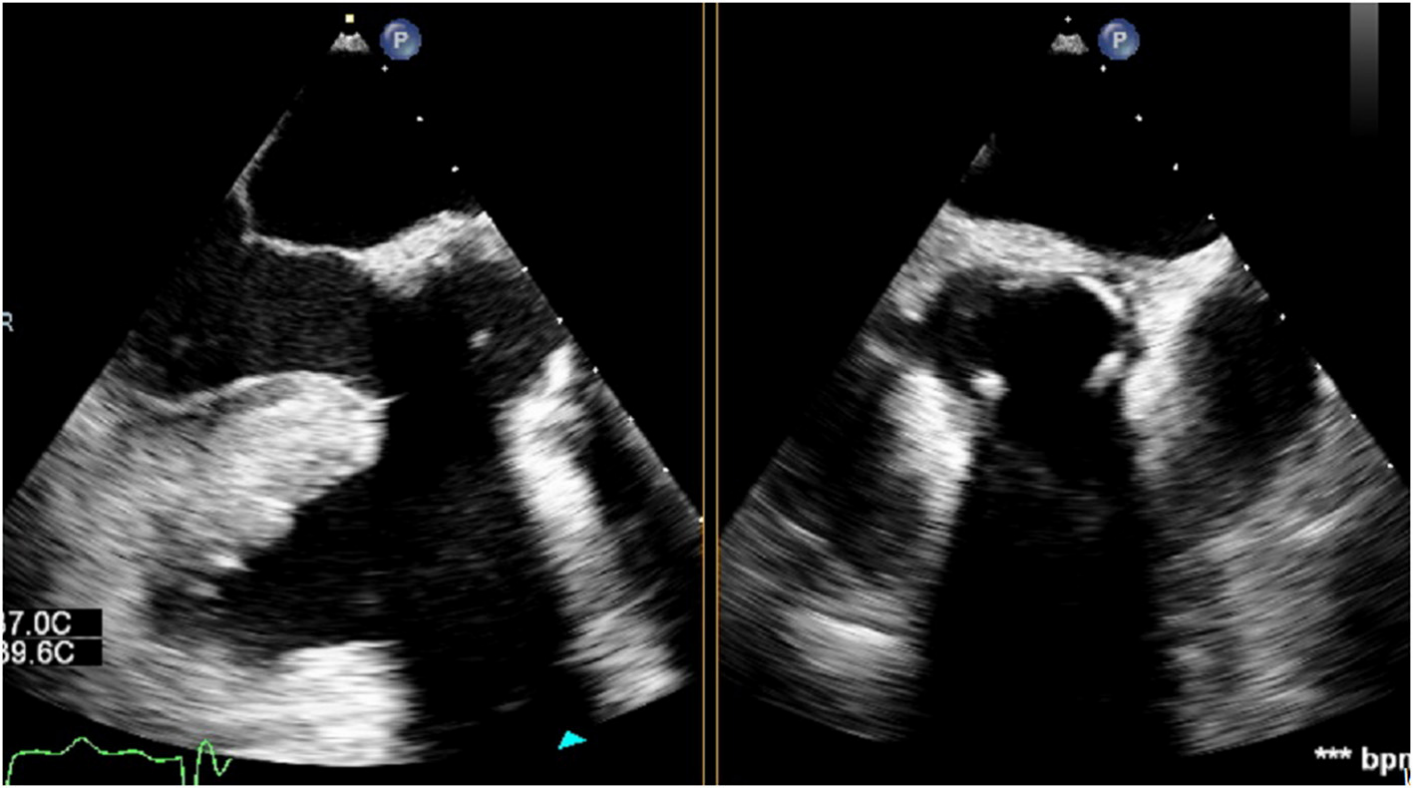
Sub-annular stainless steel frame casting an acoustic shadow which obscures the contralateral side of the valve.

After weaning from cardiopulmonary bypass, the absence of para-valvular leak (PVL) was verified on TEE. On mid-esophageal views, the stainless steel frame casts an acoustic shadow that can obscure the contralateral side of the valve (Figure 2). Therefore, trans-gastric views should be obtained to adequately assess PVL and stent expansion.

### Retrospective Measurement of Native LVOT Diameter at the Landing Site of the Sub-annular Stent

**Figure 3:**
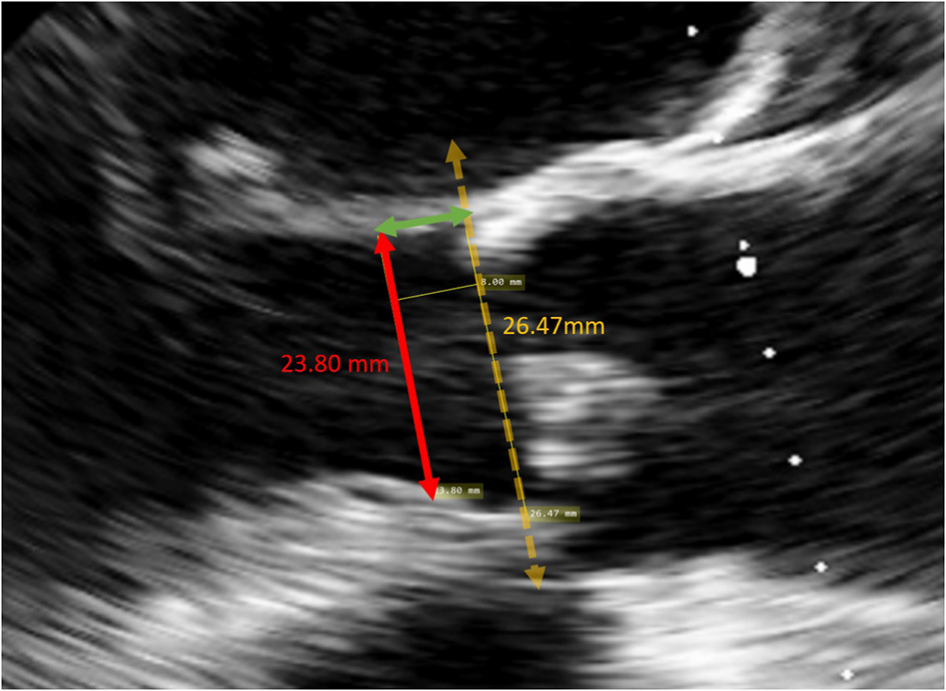
Measurement of the LVOT diameter at the landing site of a size 25 valve. A line was drawn from the inner edge of the septal endocardium to the inner edge of the anterior mitral leaflet in mid-systole, parallel to the aortic annulus, 8 mm apical to the annulus.

The LVOT diameter at the landing site of the sub-annular stent was retrospectively measured using the archived TEE images. In the mid-esophageal long-axis view, a line was drawn from the inner edge of the septal endocardium to the inner edge of the anterior mitral leaflet in mid-systole, parallel to the aortic annulus (Figure 3). The theoretical landing site of the sub-annular stent would be 6.0, 6.6, 7.2 or 8.0 mm apical to the aortic annulus depending on the valve size and the corresponding stent length.

**Figure 4:**
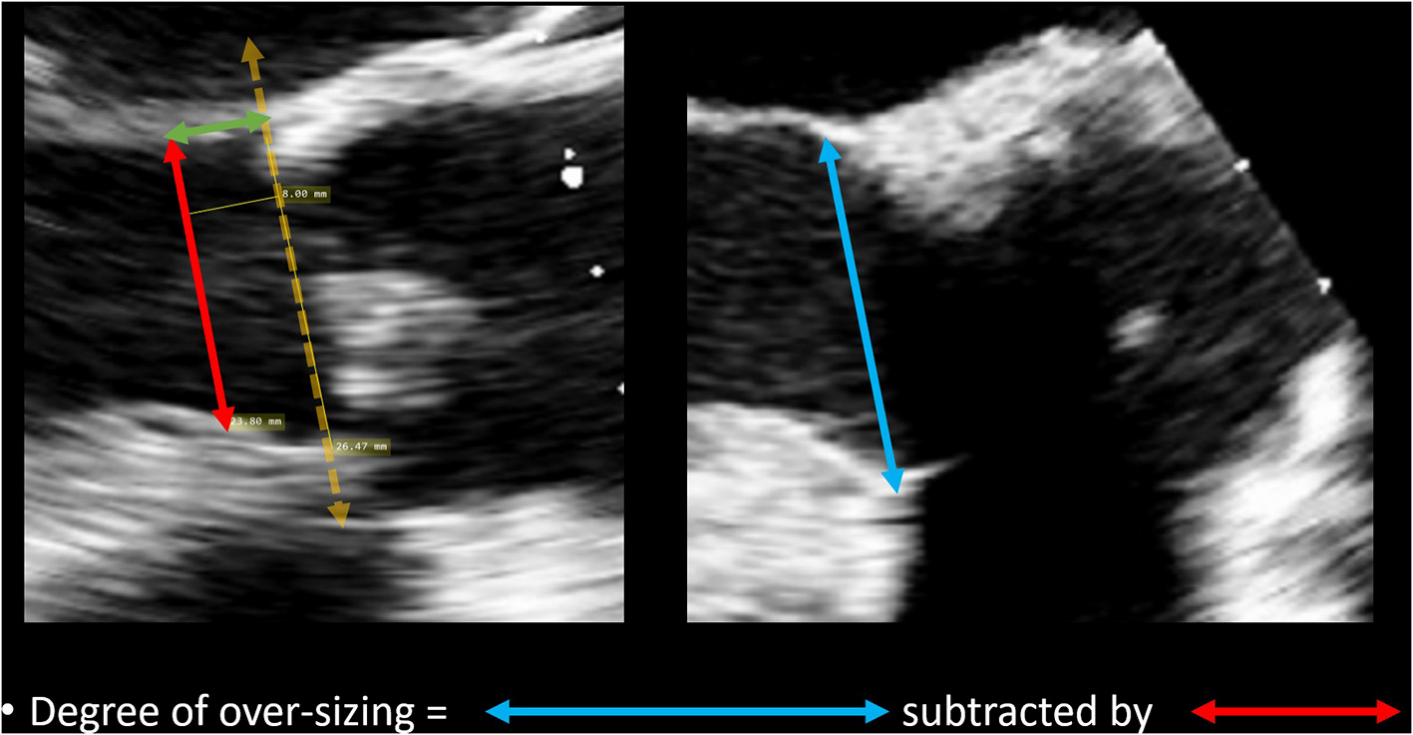
Method of calculating degree of over-sizing. The diameter of the sub-annular stent was subtracted by the native LVOT diameter.

The degree of over-sizing was calculated by subtracting the measured native LVOT diameter by the external diameter of the sub-annular stent from manufacturer specifications (Figure 4).

Relevant echocardiographic parameters including aortic annular diameter, the presence of septal bulge (defined as basal-septal wall thickness at least 2 mm thicker than the mid-septal wall thickness), and the final diameter of the sub-annular stent at its landing site were also analyzed.

### Follow-Up

All patients received round-the-clock telemetry monitoring until the day of discharge. 12-lead electrocardiograms were performed on post-operative days 2 and 14, and during their first clinic visit in the third post-operative month. Transthoracic echocardiogram (TTE) was performed for all patients in the third post-operative month.

The primary endpoint was new onset conduction disturbance, which was defined as the occurrence and non-resolution of new onset bundle branch block or complete heart block by post-operative day 90. Our unit policy was to stop all atrioventricular nodal blocking agents including beta-blockers and calcium channel blockers if conduction disturbances were identified. A typical 14-day waiting period was allowed for transient conduction disturbances to resolve prior to PPM implantation.

### Statistical Methods

Descriptive statistics were reported as mean with standard deviation for continuous variables and as frequencies and percentages for categorical variables. Univariate analysis was performed to determine risk factors for new onset conduction disturbance. For categorical data, the χ^2^ test was used to evaluate univariate categorical data when the minimum number of observations in a category was over 5; otherwise, likelihood ratios G-tests were used. Student's *t*-test was used to compare means of independent samples, after verifying equality of variances with Levene's test and normality of distribution with Shapiro-Wilk Test. Variables with a *p*-value < 0.05 on univariate analysis were considered statistically significant and would be included in the multivariate predictive model with the enter method. Statistical analysis was completed using IBM SPSS Statistics 23 (IBM Corporation, Armonk, NY). TEE images were analyzed with MicroDicom viewer. Follow-up data up to 90 days were analyzed.

## Results

### Patient Demographics and Baseline Characteristics

Patient demographics and baseline characteristics were shown in Table 2. There were 20 men (66.7%) and 10 women (33.3%). Age ranged from 57 to 85 years, with a mean of 68.3 ± 6.6 years. 80% had normal left ventricular ejection fraction. 20% had NYHA III or IV heart failure symptoms.

**Table 2:** Demographics and baseline characteristics of the cohort.

**Baseline characteristics**	***n***	**%**
Female	10	33.3
NYHA III or IV	6	20.0
Hypertension	18	60.0
Diabetes mellitus	10	33.3
Atrial fibrillation	6	20.0
Pre-existing PPM	2	6.6
Extracardiac arteriopathy	2	6.6
Chronic lung disease	2	6.6
End-stage renal failure on dialysis	1	3.3

### Operative Details

Operative details were shown in Table 3. Short-term post-operative outcomes were shown in Table 4. Full sternotomy was performed in 80%, with hemi-sternotomy performed in 16.7%. Most excised valves were trileaflet. Most patients suffered from severe aortic stenosis, with a minority suffering from mixed severe AS and AR. Concomitant procedures were required in half of the cases, with the majority being CABG. Operative mortality was 3.3%.

**Table 3:** Operative details and intraoperative findings.

**Operative details**	***n***	**%**
**Valve dysfunction**		
Aortic stenosis	25	83.3
Aortic regurgitation	2	6.7
Mixed AS and AR	3	10.0
**Valve pathology**		
Trileaflet, degenerative	25	83.3
Bicuspid, degenerative	4	13.3
Old prosthetic valve dehiscence due to aortitis	1	3.3
**Surgical approach**		
Full sternotomy	24	80
Hemisternotomy	5	16.7
Right VATS	1	3.3
**Concomitant procedures**		
CABG	10	33.3
AsAo replacement	4	13.3
Mitral valve repair or replacement	3	10.0
Tricuspid valve repair	2	6.7
Root enlargement	1	3.3

**Table 4:** Post-operative complications.

**Complications**	***n***	**%**
Unsuccessful deployment	0	0
In-hospital mortality	1	3.3
PPM implantation	2	6.6
New onset BBB	2	6.6
Moderate PVL	2	6.6
Severe PVL	0	0
Resternotomy for bleeding	2	6.6
Ischaemic stroke	1	6.6
Myocardial infarction	0	0
ECMO	0	0
Acute valve thrombosis	0	0
Acute renal failure requiring renal replacement therapy	0	0
Structural valve degeneration within 3 months	0	0

### Results of Peri-procedural TEE and Follow-Up TTE

On pre-bypass TEE, septal bulge was present in one-third of the cohort. On post-bypass TEE, the sub-annular stainless steel frame diameter exceeded the diameter of the native LVOT at the landing site in all but two patients, indicating a variable degree of over-sizing in all patients. One-third of all patients received valves with sub-annular stent oversizing greater than or equal to 5.0 mm. There were no cases of moderate or severe paravalvular leak. However, TTE at post-operative 3 months identified two patients (6.7%) with moderate PVL. The mean trans-valvular gradients ranged from 2 to 15 mmHg, with a mean of 6.33 ± 3.20 mmHg.

### New Onset Conduction Disturbance and Requirement for PPM

A pre-existing permanent pacemaker was present in two patients. Atrial fibrillation was present in 20% of the cohort. New conduction disturbance occurred in 4 patients (14.3%). This included two incidences of LBBB and two incidences of PPM implantation due to complete heart block. Interestingly, both patients who required pacemakers evolved from LBBB to complete heart block in a delayed fashion at least 30 days after the index operation. The 3-month PPM rate of 7.1% compares favorably with the 13.6% 30-day PPM rate reported by the TRANSFORM trial (2).

### Risk Factors for Conduction Disturbances

The two patients who already had a pacemaker pre-operatively were excluded from the subsequent analysis. In the remaining 28 patients, relevant demographical, operative, and echocardiographic findings were analyzed using the corresponding univariate tests listed in the Statistical Methods section. The included factors were age, female sex, sub-annular frame length ≥8 mm, presence of septal bulge, true bicuspid native valve, LVOT landing site oversizing greater or equal to 5 mm, and pre-operative atrial fibrillation.

To compare the degree of oversizing between those with conduction disturbances and those without, an independent samples *t*-test was conducted. Levene's test of equality of variances was not violated [*F*_(1,25)_ = 0.185, *p* = 0.67]. This test was found to be statistically significant, *t*(25) = 2.39, *p* < 0.05, indicating that those with conduction disturbances received valves with more profound LVOT over-sizing (5.48 ± 0.50 vs. 3.36 ± 1.74 mm).

An association was identified between LVOT landing site over-sizing greater or equal to 5.0 mm and post-operative conduction disturbance, χ^2^(1, *N* = 28) = 11.67, *p* = 0.001. Results of the analysis were shown in Table 5. Kendall's tau (τb) is 0.645, indicating a strong association. Of note, when the oversizing was ≥5.0 mm, as was the case in nine patients, new conduction disturbances occurred in 44.4%. Other risk factors did not reach statistical significance.

**Table 5:** Univariate analysis of risk factors for new onset conduction disturbance.

**Risk factors**	**Likelihood** **ratio G-test**	***p*-value**
Age >60 (*n* = 20)	2.59	0.107
Female sex (*n* = 10)	0.11	0.741
Frame length ≥8 mm (*n* = 8)	2.26	0.133
LVOT landing site over-sizing >5 mm (*n* = 9)	11.67	0.001^*^
Septal bulge (*n* = 9)	0.68	0.409
True bicuspid (*n* = 4)	1.01	0.602
Pre-operative atrial fibrillation (*n* = 5)	0.16	0.687

* *p < 0.05, statistically significant*.

## Discussion

### Clinical Relevance

The Edwards INTUITY valve is a valuable addition to the armamentarium of the modern aortic root surgeon (4). As demonstrated in TRANSFORM, TRITON, and CADENCE-MIS, the valve is fast and easy to implant with consistent reduction in crossclamp times (5–7). For complex operations or patients with marginal cardiac function, reduced crossclamp times may lead to a shorter ICU length of stay (8).

Another advantage of the INTUITY valve is improved hemodynamic performance, with lower trans-valvular gradients and larger effective orifice area compared with traditional SAVR (9). In an Austrian single-center cohort analysis of 659 patients with small aortic roots who received the size 19 and 21 valves, the incidence of severe patient-prosthesis mismatch was merely 9.7% (10). The prevailing hypothesis for this observation is that the sub-annular frame forms a larger and more circular “neo-LVOT,” as demonstrated in an *ex vivo* mechanistic study by Sadri et al. (11).

In our experience, severe aortic stenosis patients with an hourglass-shaped LVOT and septal bulge benefit the most from the INTUITY valve, because traditional surgical valves cannot adequately address this sub-valvular component of aortic stenosis (12, 13). Over-sizing within the LVOT is necessary for anchorage, but excessive over-sizing may lead to conduction disturbances (14). This remains the Achilles' heel of rapid deployment valves.

In order to fully realize the benefits of improved hemodynamic performance, conduction disturbances must be avoided. We believe the key lies with accurate sizing. Unique to the INTUITY valve is the fact that the depth of implantation is pre-determined by the frame length of each valve size. As a result, the diameter of the landing site within the native LVOT can be sized pre-operatively.

There are numerous pitfalls associated with intraoperative sizing the INTUITY valve. First, one should theoretically size the native LVOT and not the annulus, because the LVOT is the site of anchorage. Second, the sub-annular stent cannot be sized *in vivo*, because the valve sizers corresponded to the sewing ring and not the stent. Thus, the magnitude of radial expansion of the circular frame within the elliptical LVOT cannot be assessed until after the crossclamp is taken off, by which time damage to the conduction bundle would have already occurred. Third, unlike planning for TAVR, ECG-gated multiplanar CT assessment of the aortic root is not a mandatory component of pre-operative SAVR assessment. Valve sizing is performed purely using valve sizers, based on the “snugness” with which they pass through the annulus – a process which relies heavily on surgeon experience. If a pre-bypass TEE measurement is absent, surgeons can only follow their intuition in the choice of valve size.

Our results demonstrated that pre-procedural TEE sizing of the LVOT can guide subsequent choice of valve size and potentially prevent conduction disturbances. The degree of “safe” over-sizing is around 3 mm, as demonstrated in patients without conduction disturbances. Although over-sizing is inevitable to anchor the sub-annular stent, over-zealous over-sizing beyond 5 mm should be avoided. For patients with narrow LVOT in whom oversizing beyond 5 mm is required, it may be prudent to avoid implanting an INTUITY valve implantation and opt for traditional sutured SAVR or septal myectomy.

### Limitations

The sample size was small, and any findings generated were at best hypothesis generating. The study was retrospective in nature and had inherent weaknesses including selection bias. As with any surgical device, learning curve has a significant influence on outcomes including the choice of valve size.

The major weakness, however, remained interobserver differences and measurement errors in annular diameter and hemodynamic performance using TEE alone, due to its inferior spatial resolution when compared with CT. In fact, in a prospective study comparing aortic annular measurements from three-dimensional TEE and CT prior to implantation of the first-generation CoreValve, TEE measurements were on average 9% smaller than that of CT, and the degree of concordance with regard to the final choice of valve size was merely 44% (15). A similar finding was observed in SAPIEN valves as well (16).

Two key differences between TAVR and INTUITY valves may limit the applicability of these findings to the present study. First, the depth of implantation for TAVR valves is variable. As such, it is difficult to predict the exact LVOT landing site from the pre-operative CT. Second, anchorage of TAVR valves invariably involve both the annulus and LVOT, whereas sealing and anchorage for the INTUITY valve is solely dependent on the LVOT.

Although contrast CT assessment will undoubtedly provide more precise measurements of the left ventricular outflow tract, availability was hampered by resource constraints in our locality. Further investigations into the degree of concordance between TEE and CT measurements of the LVOT in INTUITY valves may be a worthwhile avenue for research.

## Conclusion

During INTUITY valve implantation, in addition to the aortic annulus, the landing site of the sub-annular stent within the native LVOT should also be sized pre-bypass. Over-sizing the native LVOT by 5 mm or more was associated with an increased risk of new onset conduction disturbances and should be avoided.

## Data Availability Statement

The raw data supporting the conclusions of this article will be made available by the authors, without undue reservation.

## Ethics Statement

The studies involving human participants were reviewed and approved by Joint Chinese University of Hong Kong—New Territories East Cluster Clinical Research Ethics Committee. Written informed consent for participation was not required for this study in accordance with the institutional requirements.

## Author Contributions

KL, TF, and RW: concept or design. KL, YH, SC, and AL: acquisition of data. KL, YH, SC, TF, AL, and RW: analysis or interpretation of data. KL, YH, SC, TF, and RW: drafting of the manuscript. All authors contributed to the article and approved the submitted version.

## Conflict of Interest

The authors declare that the research was conducted in the absence of any commercial or financial relationships that could be construed as a potential conflict of interest.

## Abbreviations

AF: atrial fibrillation
AVR: aortic valve replacement
CHB: complete heart block
CI: confidence interval
LBBB: left bundle branch block
LVOT: left ventricular outflow tract
PPM: permanent pacemaker
PVL: paravalvular leak
RBBB: right bundle branch block
RD-AVR: Rapid deployment aortic valve replacement
SAVR: Surgical aortic valve replacement
TAVR: transcatheter aortic valve implantation
TEE: trans-esophageal echocardiogram
TTE: trans-thoracic echocardiogram.
